# Right Atrial Myxoma Removal Followed by COVID-19 Infection and Possibly Related Late Pericardial Effusion in a 31-Year-Old Male

**DOI:** 10.7759/cureus.72498

**Published:** 2024-10-27

**Authors:** Yossy Machluf, Majd Said, Daniel L Fink, Yigal Chechik, Yoram Chaiter

**Affiliations:** 1 Medical Corps, Israel Defense Forces, Rehovot, ISR; 2 Medical Corps, Israel Defense Forces, Haifa, ISR; 3 Cardiology: Pediatric Cardiology Unit, HaEmek Medical Center, Afula, ISR; 4 Medical Corps, Israel Defense Forces, Tel Hashomer, ISR

**Keywords:** cardiac surgery, covid-19, pericardial effusion, pericardiotomy, right atrial myxoma

## Abstract

Myxoma of the heart is the most common cardiac neoplasm in adults, typically originating in the left atrium. Despite being benign, these tumors can cause significant local mechanical disturbances and impair cardiac function. Surgical removal of the myxoma is usually necessary to prevent potentially severe complications such as embolization. Cardiac myxoma can occur sporadically or as part of a familial condition, notably inherited with autosomal dominance in the Carney complex (approximately 10% of cases). The clinical presentation of these tumors varies, ranging from asymptomatic cases found incidentally during cardiac echocardiograms performed for unrelated reasons, to severe cardiovascular events such as heart failure, stroke, pulmonary emboli, or sudden death. We describe the case of a previously healthy 31-year-old male who presented with chest pain, which was later found to be associated with a right atrial myxoma. One and a half years after successful surgical removal, he developed acute pericarditis following a bout of COVID-19, which was effectively treated with nonsteroidal anti-inflammatory drugs (NSAIDs) and colchicine. While post-COVID-19 pericarditis is a recognized condition, we suspect that our patient was predisposed to this complication due to his previous cardiac surgery and pericardiotomy, which are well-known risk factors for pericarditis.

## Introduction

Cardiac myxomas are the most common primary cardiac tumors, typically arising from the left atrium [1-3]. They are rare benign neoplasms arising from multipotent mesenchyme characterized by lipidic cells in a vascular myxoid stroma [4]. Clinical presentations vary widely from asymptomatic to typical influenza-like symptoms to heart failure, stroke, and even cases of sudden death with a postmortem diagnosis. Common presentations include constitutional symptoms, embolic or cardiac symptoms, systemic manifestations (fever and sweating), dyspnea, and neurological issues [1,5,6]. Comorbidities such as hypertension, diabetes, and hyperlipidemia are frequently observed in patients with atrial myxoma. Although not related to our case, it is interesting to note that among patients with coronavirus 2019 (COVID-19), atrial myxoma can present asymptomatically or with various, and even atypical, symptoms [7,8]. This makes its diagnosis and management more challenging, necessitating multimodality cardiac imaging such as transthoracic echocardiogram and cardiac MRI. In general, echocardiography is the primary diagnostic tool for atria myxoma, followed by CT and MRI confirmation. The macroscopic appearance of the myxoma can be solid or papillary, with the solid type being more common. Histo-pathologically, myxoma shows positivity for endothelial cell markers such as CD31 and CD34. Prompt surgical resection is the preferred treatment, resulting in an excellent prognosis with low mortality and recurrence rates. Long-term survival rates after myxoma removal are comparable to the general population [1,9].

The annual incidence of atrial myxoma ranges from 0.5 [10] to approximately 10 cases per million people [6]. It most commonly occurs in the fifth decade of life, predominantly in middle-aged to elderly individuals, with a female predominance [3,11-13], although some studies have reported a male predominance [6]. The left atrium is involved in 75% to over 90% of all cases, with rare cases involving the septum or the ventricles [9,11]. Symptoms of right atrial myxoma may initially be vague, such as intermittent or persistent fever, weight loss, chronic anemia, and joint pain. It can be misdiagnosed until serious medical issues develop such as pulmonary hypertension from chronic emboli of tumor fragments or Budd-Chiari syndrome causing abdominal pain due to obstruction of flow to the right ventricle. Rarely, cases of myocardial tamponade or infected right atrial myxoma have been reported [14]. While most cases are sporadic, there are also familial variants such as Carney complex syndrome [2,4]. This is a multisystem tumorous disorder characterized by myxoma of the heart, skin, and breast, spotty skin pigmentation (lentigines and blue nevi), endocrine tumors (adrenal, testicular, and pituitary), and peripheral nerve tumors (schwannomas). The condition is inherited as an autosomal dominant trait. The most serious components of the syndrome are cardiac myxoma and psammomatous melanotic schwannoma [15].

We report a case of a young male patient with a right atrial myxoma. He initially experienced atypical, non-specific chest pain, nausea, and shortness of breath. About eighteen months after cardiac surgery and pericardiotomy, he developed pericardial effusion following a symptomatic COVID-19 infection, despite having received three COVID-19 vaccinations.

## Case presentation

Patient background

A 31-year-old male patient presented to the Emergency Ward in August 2020 with symptoms including nausea, non-specific chest pain, weakness, and sweating. He had no previous history of heart disease, and his family history was unremarkable. His past medical conditions included knee ligament problems, low back pain with discopathy, gastroesophageal reflux, and *Helicobacter pylori* infection. He used contact lenses and had a known sensitivity to Ofloxacin. In childhood, he underwent surgical correction of an undescended testicle and an appendectomy. He had no endocrine conditions, skin lesions, or pigmentations.

Diagnostic workup

After being evaluated in the Emergency Ward, he was found to have normal cardio-respiratory function with no other complaints. He had no fever (36.8 ˚C), his blood pressure was 132/74, pulse 102, oxygen saturation 100%, and his physical examination was normal. His troponin levels (repeated twice) were normal. He was discharged home with a recommendation to undergo an ambulatory echocardiogram, stress test, and cardiology consultation.

**Figure 1:**
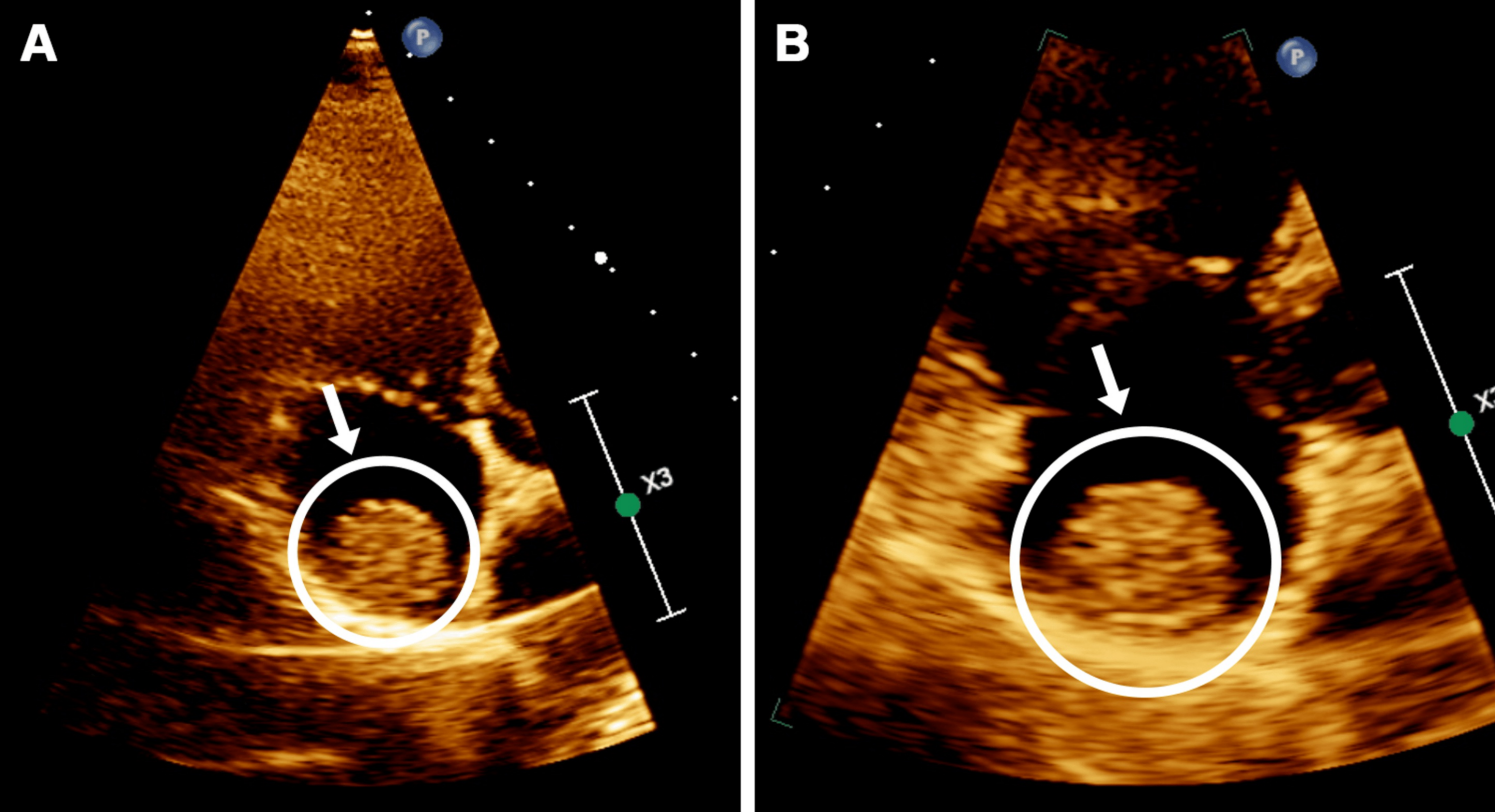
Echocardiogram image of the right atrial myxoma before surgical removal (August 2020)

His echocardiogram exam revealed a mass measuring 1.8 cm in diameter attached to the posterior wall of the right atrium, not related to the vena cava or coronary sinus (Figure 1).

Surgical intervention

**Figure 2:**
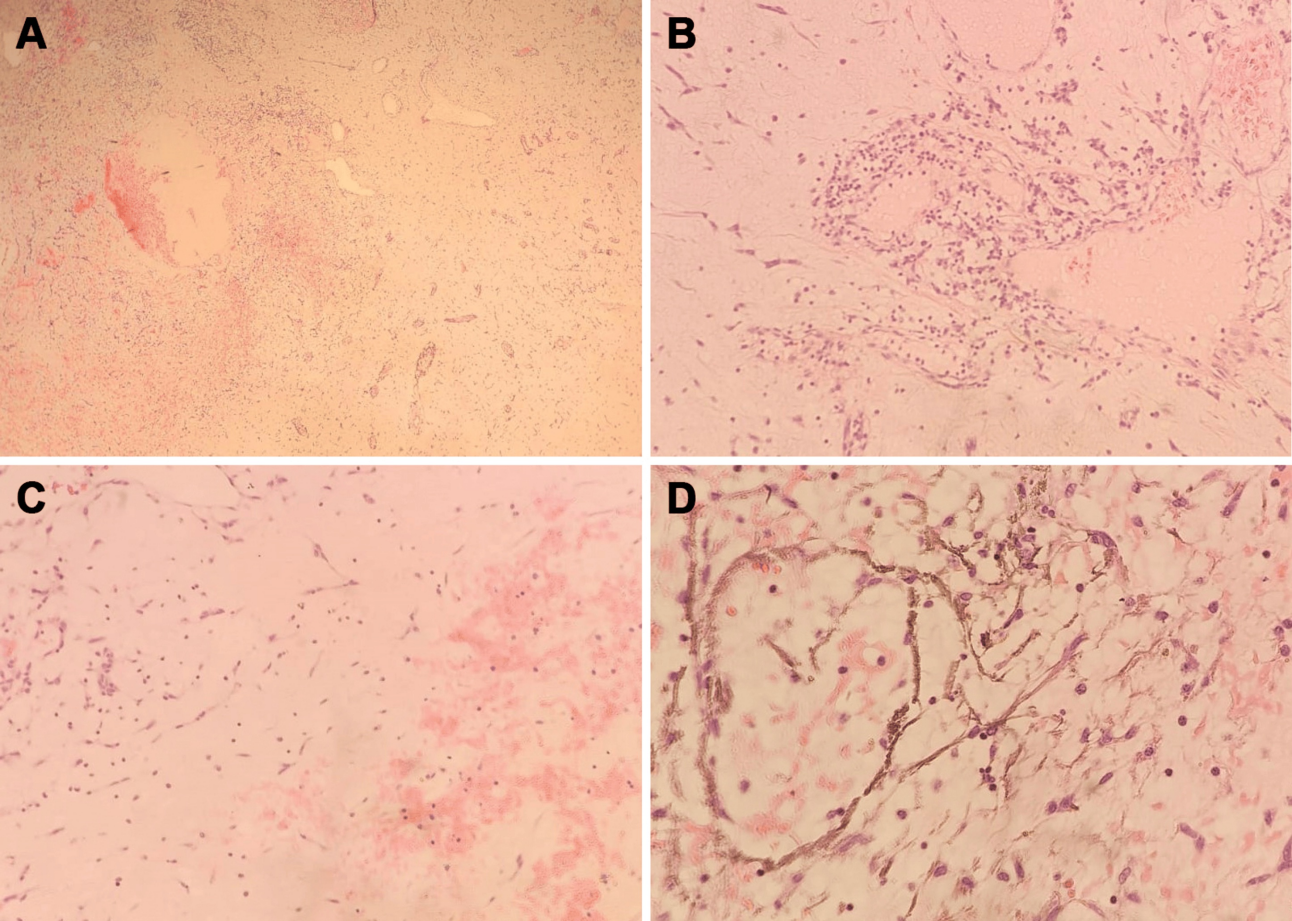
Hematoxylin and eosin staining of the tumor cells and surrounding tissue (August 2020) (A) Myxoid stroma with tumor cells, blood vessels, hemorrhages, and hemosiderin deposits (magnification: x100); (B) Polygonal tumor cells, forming cords and nests, surrounding vessels (magnification: x200); (C) Cords of tumor cells, inflammatory cells, and hemorrhage (magnification: x200); (D) Hemosiderin deposits at higher magnification (magnification, x400)

The patient had cardiac surgery on August 8, 2020. Pre-surgery blood tests showed slightly elevated total bilirubin levels. Serological tests were negative for *Toxoplasma gondii* antibodies and cytomegalovirus (CMV) immunoglobulin M (IgM) but positive for CMV IgG and Epstein-Barr virus nuclear antigen (EBNA). The tumor was successfully removed, and the histology results showed a gelatinous mass measuring 2.5 centimeters in diameter attached to a 0.6-centimeter muscle fragment. It was identified as a myxoma tumor with clear surgical margins (Figure 2).

Postoperative course

The patient had a smooth recovery with no incidents. On the day he was discharged, he was stable with no cardio-respiratory issues, normal blood test results, and a chest X-ray that showed a small improving post-surgical pneumothorax on the right side. His echocardiogram was completely normal, showing a small right atrium and no valvar disease.

Three weeks after the surgery, the patient had a follow-up at the cardiothoracic surgery clinic. He had recovered quickly and was walking up to a kilometer every day. His wound was normal, and he had stopped taking all medications. A chest X-ray showed no pneumothorax. A month after the surgery, his echocardiogram was normal. He returned to work within three months, experiencing typical postoperative pain but with a well-healing mid-sternal scar. During a follow-up echocardiogram three months after the surgery, a small area was seen in the right atrium, consistent with scarring post-mass removal, with no evidence of tumor recurrence. A further follow-up chest X-ray was normal.

During the COVID-19 pandemic, he received three doses of the vaccine but later developed clinical disease in March 2022, 1.5 years post-surgery, and had a typical recovery.

Follow-up

Two months later, in May 2022, the patient returned to the Emergency Ward with chest pains. Initial blood tests showed increased CRP at 58 mg/L while other results were normal. Despite the recommendation for hospitalization, the patient declined, and his CRP levels later decreased to 46 mg/L. He continued to experience right chest pain that radiated to the right shoulder and hand. During a physical exam, no abnormal heart sounds or murmurs were detected, including no pericardial rub, and there were no signs of infection, such as redness and discharge, or swelling in the scar area. A lung examination revealed no pathological findings. Tenderness and muscle spasms were observed around the right medial scapula, and there was a mild restricted range of motion in the right shoulder due to pain. Additionally, a weakening of the radial pulse was felt when raising the right hand and turning the head. The ECG showed no signs of acute ischemia or arrhythmias, but there was a double hump in P waves in leads V2-V3.

The patient was then referred to the emergency ward, where his CRP levels were again found to be 58 mg/L, but troponin tests came back negative, and chest X-rays showed no congestion or infiltrate. His ECG revealed normal sinus rhythm at 114 beats per minute without signs of ischemia. An echocardiogram indicated a mild-to-moderate pericardial effusion with no abnormal wall motion. He agreed to be admitted to the cardiology department for observation and was treated with colchicine and nonsteroidal anti-inflammatory drugs (NSAIDs). After treatment, he was discharged with a prescription for ibuprofen and colchicine for a three-month course.

Soon after starting anti-inflammatory treatment, the patient's pericarditis resolved. A follow-up echocardiogram in September 2022 showed no recurrent mass in the right atrium or pericardial fluid. He was able to return to his full work schedule after three weeks. He has remained asymptomatic for two years. A subsequent echocardiogram in June 2024 did not reveal tumor recurrence or pericardial effusion.

**Figure 3:**
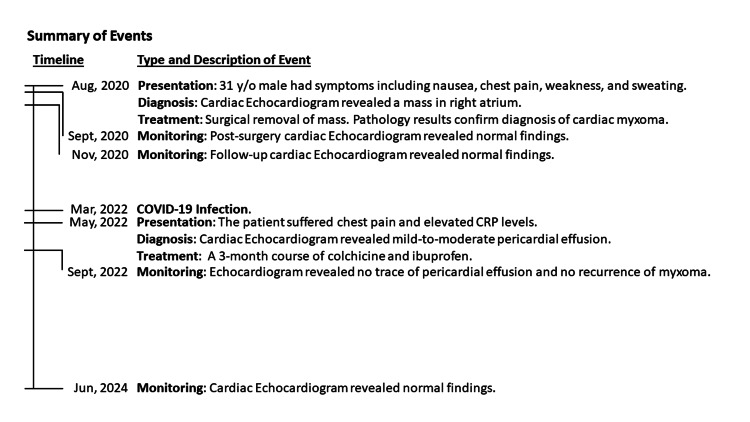
A schematic chronological summary of the main events of the case study

The overall chronological medical course of this case report is summarized in Figure 3.

## Discussion

Cardiac myxomas are the most common benign tumors of the heart [1-3] and can present with a wide range of symptoms, from no symptoms to severe cardiorespiratory and neurological issues, depending on the location of the tumor. Embolization of myxomas can cause vascular and other complications such as stroke [1,5,6]. We present a relatively rare case of a young male with a right-sided atrial myxoma, which was removed by pericardiotomy. There was no evidence of pericardial effusion for over 18 months after surgery; however, approximately 1.5 months after COVID-19 infection, a pericardial effusion appeared and was successfully treated with colchicine and NSAIDs.

The etiology, pathophysiology, and implications of pericardial effusion have been reviewed recently [16]. In short, it is characterized by the accumulation of excess fluid within the pericardial sac, and it can be classified based on its onset, size, and composition of fluids. Infections, injuries, and other medical conditions are among the leading underlying causes of pericardial effusion. Fluid accumulation may lead to elevated pressure, mainly on the right side of the heart, impairing its function, which in severe cases can cause cardiac tamponade, a life-threatening medical emergency. Post-pericardiotomy pericardial effusions are known complications of cardiac surgery, typically occurring within the first few weeks after the procedure [17]. Cardiac surgery can lead to pericardial changes such as scarring and possible inflammation [15]. In the present case, pericardial effusion occurred almost two years after right atrial myxoma removal.

Pericardial effusions, alongside other cardiac abnormalities, are also described as a complication of COVID-19 infection [18-20]. COVID-19 can trigger inflammatory processes that may lead to pericardial effusion. We speculate that individuals who have undergone cardiac surgery prior to a COVID-19 infection may be more susceptible to developing subsequent pericardial effusions following the infection compared to those without a history of cardiac surgery. Since there was no aspiration and subsequent analysis of the pericardial fluid, crucial information on the underlying process was not obtained. For example, bloody pericardial effusion may be caused by iatrogenic (including the effect of anticoagulant therapy, trauma, post-invasive cardiac procedures, etc.), malignancy, atherosclerotic heart disease (mainly complications of acute myocardial infarction), tuberculosis (one of the most common causes of pericarditis/pericardial effusion in Africa and developing countries), and idiopathic reasons. Milky fluid may indicate the involvement of the lymphatic system while cloudy and turbulent fluid may suggest increased capillary leakage and leukocytosis and is concerning for infectious effusion. We acknowledge that the lack of this analysis represents a significant gap in the understanding of this case. Nevertheless, since the fluids of both post-pericardiotomy syndrome and COVID-19 might show only general inflammatory properties within the pericardium, such as an elevated WBC level and low pH and glucose, it most likely would not have contributed further to clarify the exact nature of the case, and to our understanding if and which of the two conditions was involved in the pericardial effusion pathogenesis.

To the best of our knowledge, cases involving a post-pericardiotomy state, COVID-19, and subsequent late pericardial effusion have not been reported to date. It remains to be seen whether further reports will support an association between these conditions and will help shed light on a possible underlying mechanism.

## Conclusions

We presented a rare case of a young male with a right-sided atrial myxoma that was successfully resected. There were no signs of pericardial effusion for over 18 months after pericardiotomy. However, approximately 1.5 months after COVID-19 infection, the patient developed a pericardial effusion, which was effectively treated with colchicine and NSAIDs. Post-pericardiotomy pericardial effusions typically occur a few weeks after cardiac surgery. However, in this case, it occurred late after the surgery following the COVID-19 infection. Pericardial effusions are described as a complication of COVID-19 infection, possibly signifying an association with generalized inflammation. The possibility that individuals who have had cardiac surgery before contracting COVID-19 may be more prone to developing subsequent pericardial effusions requires further investigation.
